# Effects of Temperature-Moisture Interactions on Storage Survival and Virulence in Two Entomopathogenic Nematode Species

**DOI:** 10.3390/insects17070723

**Published:** 2026-07-13

**Authors:** Hongyan Li, Kexin Zhang, Tingwei Zhang, Xiujuan Qian

**Affiliations:** 1 Biocontrol Engineering Laboratory of Crop Diseases and Pests of Gansu Province, College of Plant Protection, Gansu Agricultural University, Lanzhou 730070, China; lihongyan2025@163.com (H.L.); zhangtw@gsau.edu.cn (T.Z.); 2 Wuwei Shiyanghe Forestry General Farm, Wuwei 733000, China; 3 State Key Laboratory for Biology of Plant Diseases and Insect Pests, Institute of Plant Protection, Chinese Academy of Agricultural Sciences, Beijing 100193, China; zhangkx199404@163.com

**Keywords:** *Heterorhabditis megidis* 0627M, *Steinernema feltiae* 0619HT, storage method, temperature, moisture content, survival rate, virulence

## Abstract

Entomopathogenic nematodes (EPNs) are among the most promising biocontrol agents; however, their short shelf life constrains commercial application. This study evaluated the effects of storage temperature (6 °C and 25 °C) and sponge substrate moisture content (42%, 48%, and 55%) on the 18-week survival and post-storage virulence of two indigenous EPN species from Gansu Province, China: *Heterorhabditis megidis* 0627M and *Steinernema feltiae* 0619HT. A Generalized Linear Mixed Model (GLMM) revealed that storage duration, temperature, moisture content, and species all significantly affected survival of *H. megidis* 0627M and *S. feltiae* 0619HT (all *p* < 0.001). Low-temperature storage (6 °C) reduced mortality odds by 82.4% compared with room temperature (OR = 0.176, 95% CI: 0.117–0.263, *p* < 0.001). High moisture content (55%) increased mortality odds by 10.1-fold relative to moderate moisture (48%; OR = 10.114, 95% CI: 6.155–16.618, *p* < 0.001), whereas low moisture (42%) showed no significant difference from 48% (OR = 0.942, *p* = 0.810). The two species exhibited distinct adaptation strategies: *S. feltiae* 0619HT achieved the highest survival under low-temperature storage at 48% moisture content (56.48% at week 18), whereas *H. megidis* 0627M demonstrated a delayed competitive advantage under room-temperature, low-moisture conditions. Virulence assays revealed that low-temperature storage better preserved infectivity under most conditions. Notably, survival and virulence were not always concordant, necessitating their evaluation as complementary metrics. Species-specific storage protocols are proposed, providing a scientific basis for the future development of regionally targeted and cost-effective native EPN formulations, as well as for regionally targeted biocontrol applications.

## Simple Summary

Chemical pesticides pose well-documented risks to environmental health and food safety, thereby creating an increasing demand for sustainable alternatives. Entomopathogenic nematodes infect and kill insect pests without harming people, animals, or the environment. However, keeping these living creatures alive and effective long enough to reach farmers remains a challenge. This study explored whether simple adjustments to the storage temperature and moisture content of the packaging material could extend their useful shelf life. Two types of beneficial entomopathogenic nematodes, both collected from Gansu Province in northwestern China, were stored in a sponge at low and room temperatures under varying moisture levels for 18 weeks. Low temperature made a dramatic difference, cutting mortality by more than 82%, while excessive moisture was highly damaging. The two nematode species responded differently: one thrived at low temperature with moderate moisture, retaining over 56% survival after 18 weeks, while the other coped better at room temperature under drier conditions. Notably, higher survival did not always translate into better pest-killing ability—suggesting that counting survivors alone is insufficient for judging product quality. These findings support the development of species-specific storage strategies and provide a scientific basis for improving the formulation and application of entomopathogenic nematodes in sustainable agriculture.

## 1. Introduction

Entomopathogenic nematodes (EPNs) are obligate insect parasites belonging the families Steinernematidae and Heterorhabditidae [1]. Compared to traditional chemical pesticides, EPNs possess significant advantages, including a broad host range, strong host-seeking ability, and high efficacy against soil-dwelling and cryptic pests. They are also considered environmentally safe, with minimal impacts on non-target organisms and a low risk of resistance development [2]. In addition, infective juveniles (IJs) can be produced at relatively low cost, facilitating their application in biological control programs [3,4]. Consequently, research on EPNs and their commercialization has garnered widespread global attention [5].

*Heterorhabditis megidis* is widely distributed across temperate regions of Europe, North America, Japan [6,7], and Asia [8]. It predominantly parasitizes soil-dwelling Coleoptera, including the Colorado potato beetle and scarabaeid white grubs [9,10], and has also demonstrated efficacy against semi-subterranean Lepidoptera, such as *Mamestra brassicae* [11]. *Steinernema feltiae* exhibits a broader host range, infecting species within Diptera (e.g., *Delia radicum*) [12], Lepidoptera (e.g., *Dendrolimus pini*, *Mamestra brassicae*) [11,13,14], and Thysanoptera [15]. It is one of the most widely distributed EPN species worldwide, with confirmed native populations across Europe [15,16,17], Asia [8,18,19], and other continents, including Africa and South America [6]. Commercial formulations of both species have been predominantly developed for temperate conditions, and storage protocols tailored to indigenous strains from arid regions, such as northwestern China, remain largely uncharacterized [20,21].

Progress in EPN research has accelerated in recent years, encompassing both fundamental studies and field applications [22]. Commercial production has been achieved in several countries, with applications in turf management [23], fruit orchards [24], greenhouse vegetables [25,26], public health pests [27], rice paddies [28], suppression of soil-dwelling pests [29,30,31,32,33,34], management of boring pests [35,36], fruit tree pests [24], and wood-boring insects [37]. Despite these advances, large-scale commercialization remains limited, mainly due to difficulties in maintaining IJ viability during storage and transportation. As a non-feeding developmental stage, IJs rely entirely on endogenous energy reserves; therefore, environmental conditions that accelerate energy consumption or induce cellular damage can rapidly reduce survival [38,39]. Among abiotic factors, temperature and moisture content are widely recognized as the primary determinants of IJ survival. Elevated temperatures increase metabolic rates and energy depletion, whereas excessive desiccation disrupts membrane integrity and physiological homeostasis, ultimately resulting in mortality [40,41,42,43].

Although recent technological advances, including anhydrobiosis induction, microencapsulation, and specialized protective formulations, have successfully extended the ambient-temperature shelf life of certain commercialized EPN species [44,45,46,47], these approaches are often associated with increased production costs and limited applicability across different species. In particular, the performance of such formulations has typically been validated for a limited number of widely commercialized species—primarily *Steinernema carpocapsae*, *S. feltiae*, and *Heterorhabditis bacteriophora*. As a result, storage optimization for regionally indigenous species remains uncharacterized [22]. Consequently, the development of cost-effective, regionally adaptable storage strategies based on simple carrier materials remains a critical and unmet need.

Currently, the predominant approach for extending EPN shelf life involves the use of carrier materials to create a moderately dehydrating environment that induces IJs into a state of quiescence or diapause [44,48]. Among available carrier materials, sponge substrates have demonstrated particular promise owing to their low cost, operational simplicity, and consistent performance [49]. However, storage efficacy is strongly influenced by the interactions among nematode species, carrier properties, and the temperature–moisture parameter space. Critically, the two factors do not act independently: the most favorable moisture level for IJs’ survival shifts with temperature, as the balance between microbial competition, desiccation stress, and metabolic suppression is temperature-dependent [50,51]. This interaction has been documented in commercialized species, yet its magnitude and directionality in indigenous strains adapted to specific climatic regimes remain poorly understood.

Beyond species selection, increasing evidence suggests that locally adapted EPN populations may possess distinct stress-tolerance profiles shaped by the selective pressures of their native environments [21,52]. Gansu Province, located in northwestern China, is characterized by low precipitation and substantial diurnal temperature fluctuations. This exerts strong selective pressure on native soil organisms. Indigenous EPN species isolated from Gansu soils may have developed physiological adaptations associated with tolerance to desiccation and thermal stress, potentially providing advantages for long-term storage under comparable environmental conditions [8]. Nevertheless, systematic evaluations of these indigenous species under simulated practical storage and transportation conditions remain limited. Determining the optimal combination of temperature and moisture content that synergistically maintains long-term survival and infectivity is therefore essential for translating these local EPN resources into practical biocontrol applications.

To establish a practical storage strategy for indigenous EPN resources from Gansu Province, this study selected two representative locally isolated strains, *H. megidis* 0627M and *S. feltiae* 0619HT, as experimental subjects. The effects of different storage temperatures and moisture-content gradients on the long-term survival and virulence of these EPNs were systematically evaluated using sponge substrates under simulated practical storage conditions. This study aimed to quantify survival dynamics over an 18-week storage period and evaluate post-storage virulence against *Galleria mellonella*, with particular emphasis on determining the relationship between survival and virulence responses across different treatments. The findings provide a scientific basis and technical support for the commercial formulation, storage, and transportation of indigenous EPN products, thereby facilitating their practical application in sustainable regional green agriculture.

## 2. Materials and Methods

### 2.1. Experimental Materials

Test Insects: Fifth-instar larvae of *G. mellonella* were obtained from a commercial supplier. Upon arrival, only actively moving, uniformly sized fifth-instar larvae (weighing approximately 200–250 mg) with no visible signs of melanization or disease were selected for experiments to ensure consistent host susceptibility and minimize experimental variation.

Entomopathogenic Nematode Species: *H. megidis* 0627M (originally isolated from Tianshui, Gansu Province) and *S. feltiae 0619HT* (originally isolated from Baiyin, Gansu Province) were provided by the Ecology Laboratory of Gansu Agricultural University [8]. Both species had been maintained through periodic in vivo propagation in *G. mellonella*. Before experiments, IJs were propagated using the White-Trap method (see Section 2.2.1), and only freshly emerged IJs (≤5 days post-emergence) with >95% motility (assessed by microscopic observation) were used for storage trials. The initial IJs concentration of storage suspensions was standardized to 2 × 10^3^ IJs/mL, and all storage experiments were initiated within 24 h of IJs harvest to minimize pre-experimental aging effects.

Main Experimental Materials: The polyurethane sponge blocks were uniformly cut into small cubic pieces with a volume of approximately 0.5 cm^3^ (density: 8.25 × 10^−3^ g/cm^3^), Petri dishes (9 cm diameter), sterilized filter paper (9 cm diameter), 50 mL screw-cap centrifuge tubes, and pipette tips. All materials were sterilized at 121 °C for 30 min before use.

Major Instruments: A stereoscopic microscope (Stemi 305 LAB, Carl Zeiss Suzhou Co., Ltd., Suzhou, China), precision electronic balance (Model: BCE224-1CCN, Sartorius Scientific Instruments Co., Ltd., Goettingen, Germany), autoclave (Model: GI54DWS, Zhiwei Instruments Co., Ltd., Shanghai, China), constant temperature incubator (QRGN-880-3, Hangzhou Qisheng Electronics Technology Co., Ltd., Hangzhou, China), and pipettes with capacities of 1000 μL, 10 μL, and 1 μL (Eppendorf AG, Hamburg, Germany).

### 2.2. Experimental Methods

#### 2.2.1. Cultivation and Propagation of EPNs

**Figure 1:**
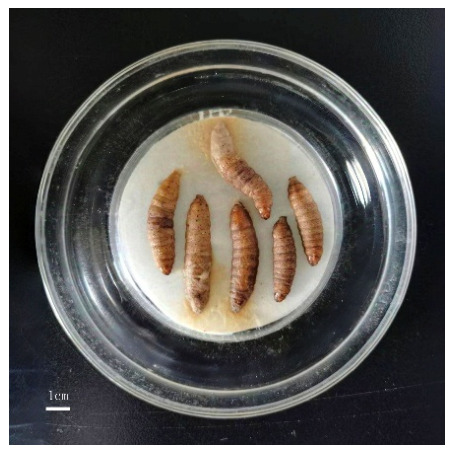
Laboratory propagation of EPNs using the White-Trap method.

All EPNs were propagated using the White-Trap method [53] (Figure 1). The culturing process was conducted under the following conditions: a 9 cm sterile Petri dish was lined with two layers of 9 cm diameter sterilized filter paper. Ten healthy last-instar *Galleria mellonella* larvae were placed into the Petri dish. Using a pipette, 800 IJs of *H. megidis* 0627M and *S. feltiae* 0619HT were applied onto the larvae. A sufficient amount of distilled water was added to keep the filter paper moist without dripping [54]. The dish was then placed in a 25 °C constant-temperature incubator in darkness. Infected larvae typically exhibited mortality within 48–72 h post-inoculation. Cadavers were transferred to modified White traps (a 9 cm Petri dish containing a smaller inverted 6 cm dish lined with moistened filter paper, onto which cadavers were placed) within 24 h of death. Traps were maintained at 25 °C in darkness, and emerging IJs were collected daily from the water reservoir for 7–10 days [55].

#### 2.2.2. Survival Assessment During Storage

Experimental design and moisture gradient preparation. The selection of moisture gradients was informed by preliminary storage trials and previously published studies on solid-carrier EPN formulations, which collectively indicate that absorbent substrates—such as vermiculite, sponge, and clay granules—create microenvironments requiring a carrier moisture content within a specific range (typically 40–60%) to maintain adequate air circulation and water balance within the pore space [56]. Moisture content below 40% VMC risks inducing excessive desiccation stress in IJs, whereas moisture content above 60% VMC may promote microbial proliferation and increase nematode mortality during storage [50]. Accordingly, three intermediate VMC levels—42%, 48%, and 55%—were selected to represent low, moderate, and relatively high moisture conditions within the practically relevant range for sponge-based storage systems. This design enabled assessment of nematode responses across a biologically meaningful moisture gradient while avoiding the detrimental effects of extreme conditions on long-term storability. Three volumetric moisture gradients were established using 50 mL screw-capped polypropylene centrifuge tubes. Sponge blocks of 0.25 g, 0.20 g, or 0.15 g were placed into individual tubes, and 22 mL of freshly harvested nematode suspension (concentration: 2 × 10^3^ IJs/mL) was added to each tube using a sterile pipette. Tubes were gently agitated for 60 s to ensure complete absorption of the suspension by the sponge matrix and uniform distribution of IJs. Volumetric moisture content (VMC) was calculated as:

$$VMC(\%)=\frac{V_{nematode\;solution}}{V_{sponge}+V_{nematode\;solution}}\times 100$$

where V nematode solution is the volume of nematode suspension (22 mL), and V sponge is the total volume of dry sponge, calculated as m sponge/ρ sponge (m sponge: total sponge mass; ρ sponge: 8.25 × 10^−3^ g/cm^3^). This yielded VMC values of approximately 42%, 48%, and 55% for the 0.25 g, 0.20 g, and 0.15 g sponge treatments, respectively. Volumetric moisture content was selected as the metric because it directly represents the proportion of water-filled pore space in the sponge matrix.

After sealing, tubes were stored in complete darkness under two temperature conditions: room temperature (25 ± 1 °C, designated RT) and low temperature (6 ± 1 °C, designated LT), maintained in constant-temperature incubators. The full experimental design comprised 2 temperatures × 3 moisture levels × 2 nematode species, yielding 12 treatment combinations. Three independent tubes were prepared for each treatment combination at each sampling time point. To prevent cross-contamination between species, all tubes were handled with dedicated sterile forceps and processed in a fixed order for each species at each time point.

Survival assessments were conducted at weekly intervals from week 0 (24 h post-setup) through week 18, yielding 19 time points per treatment. At each sampling time point, one sponge block (approximately 0.5 cm^3^) was removed from each tube using sterile forceps. Each sponge block represented a subsample of the corresponding tube rather than an independent replicate. The block was transferred to a small Petri dish containing 5 mL sterile distilled water and gently agitated for 60 s to release IJs into suspension. The resulting suspension was examined under a stereoscopic microscope at 40 × magnification. IJs exhibiting active movement and a response to mechanical stimulation were considered alive, whereas IJs showing no movement or response to stimulation were considered dead. A minimum of 100 IJs per sample was counted whenever possible. In cases where fewer individuals were recovered, all available IJs were counted. Survival was expressed as a proportion, thereby standardizing comparisons across samples despite variation in recovery numbers. No additional water was supplemented throughout the experimental period. Survival rate was calculated as:

$$Survival\;rate\left(\%\right)=\frac{Number\;of\;live\;IJs}{Total\;IJs\;counted}\times 100$$

A summary of the full experimental design, including the number of tubes per treatment combination, sponge blocks sampled per time point, and IJs counted per sample, is provided in Supplementary Table S1.

#### 2.2.3. Determination of Virulence of EPNs at Different Storage Conditions

Virulence of EPNs was assessed using last-instar larvae of *G. mellonella.* Two layers of sterilized filter paper (9 cm diameter) were placed in each Petri dish, and six healthy larvae were introduced per dish. For each treatment, the volume of the nematode suspension was adjusted according to the post-storage survival rate of recovered IJs to ensure that 300 viable, motile IJs were inoculated per dish. To evaluate the effect of storage, virulence assays were conducted for both freshly harvested IJs (≤5 days post-emergence; week 0 control) and IJs stored for 18 weeks under the experimental temperature and moisture conditions described above. Three biological replicates were established for each treatment group. All dishes were incubated at 25 °C in darkness. Larval mortality was recorded at 8 h intervals over 120 h. Death was confirmed by the absence of movement and lack of response to mechanical stimulation. Virulence was calculated as:

$$Virulence=\frac{Number\;of\;dead\;G.\;mellonella\;}{Initial\;number\;of\;G.\;mellonella}\times 100\%$$

To quantify the retention of infectivity after storage, a virulence retention index (VRI) was calculated as:

$$VRI=\frac{Virulence\;at\;week\;18}{Virulence\;at\;week\;0}\times 100\%$$

#### 2.2.4. Data Statistical Analysis

All data were processed using Microsoft Excel, and figures were generated using OriginPro 2022 (OriginLab Corporation, Northampton, MA, USA). Statistical analyses were performed using SPSS 19.0 (IBM Corp., Armonk, NY, USA). The experimental unit was defined as the individual storage tube, with three tubes per treatment combination serving as independent biological replicates. To systematically assess how storage conditions influenced survival dynamics, a Generalized Linear Mixed Model (GLMM) with binomial distribution and logit link function was employed, with the proportion of dead IJs (DeadCount/TotalCount) as the response variable. The model was specified as:

$$\begin{array}{cc} \mathrm{logit}\left(p_{ij}\right)=\beta_{0}+ & \beta_{1}\mathrm{Time}+\beta_{2}\mathrm{Temperature}+\beta_{3}Moisture+\beta_{4}Species\\ & +\beta_{5}(Time\times Temperature)\\ & +\beta_{6}(Time\times Moisture)+\beta_{7}(Time\times Species)\\ & +\beta_{8}(Temperature\times Moisture)\\ & +\beta_{9}(Temperature\times Species)+\beta_{10}(Moisture\times Species)\\ & +higher-order\;interactions+u_{i} \end{array}$$

where *p_ij_* is the probability of mortality (DeadCount/TotalCount) for tube *i* at time point *j*, *β* represents fixed-effect coefficients for storage duration (Time), temperature (Temperature), moisture content (Moisture), nematode species (Species), and all two-way, three-way, and four-way interaction terms among these factors. *u_i_* ~ N(0, σ^2^) is the random intercept for Subject ID (tube identity), included to account for the within-tube correlation arising from repeated sampling across 19 weekly time points. No random slope was specified, as the primary interest was in population-level fixed effects. Significance of fixed effects was assessed using Type III F-tests with Satterthwaite approximation for denominator degrees of freedom. Model assumptions were verified by residual diagnostics. Odds ratios (ORs) with 95% confidence intervals were derived from GLMM fixed-effect parameter estimates to quantify the magnitude and direction of each factor’s effect on nematode survival probability. Three-way factorial ANOVA, with nematode species, storage temperature, and moisture content as fixed factors, analyzed virulence data. When significant effects were detected, Tukey’s HSD test was used for post hoc comparisons to control the family-wise error rate. Simple effects analyses were conducted to further examine significant interactions.

## 3. Results

### 3.1. Comprehensive Effects of Storage Conditions on Survival Rate

GLMM analysis revealed that all main effects significantly influenced nematode survival (*p* < 0.001) (Table 1). Among these factors, temperature exerted the greatest influence on survival (*F*_(1, 661)_ = 71.139), followed by moisture content (*F*_(2, 661)_ = 56.624), species (*F*_(1, 661)_ = 29.360), and storage duration (*F*_(18, 661)_ = 8.683). These results indicate that temperature had the greatest statistical effect on survival, whereas storage duration showed a comparatively smaller effect than the other examined factors.

Among the two-way interactions, none of the interactions involving storage duration were significant, including temperature × storage duration (*F*_(18, 584)_ = 0.833, *p* = 0.662), moisture content × storage duration (*F*_(36, 584)_ = 0.204, *p* = 1.000), and species × storage duration (*F*_(18, 584)_ = 0.540, *p* = 0.939). In contrast, interactions not involving storage duration were all highly significant: temperature × moisture content (*F*_(2, 584)_ = 100.883, *p* < 0.001), species × temperature (*F*_(1, 584)_ = 38.005, *p* < 0.001), and species × moisture content (*F*_(2, 584)_ = 44.733, *p* < 0.001). These results indicate that although storage duration significantly affected overall survival, its interactions with other factors did not significantly modify the pattern of survival changes over time. Instead, the significant interaction effects among temperature, moisture content, and species identity represented the dominant factors influencing survival responses.

Among the three-way interactions, temperature × moisture content × storage duration was highly significant (*F*_(36, 492)_ = 2.143, *p* < 0.001), and species × moisture content × storage duration also showed a significant effect (*F*_(36, 492)_ = 1.538, *p* = 0.026). In contrast, species × temperature × storage duration (*F*_(18, 492)_ = 1.534, *p* = 0.074) and species × temperature × moisture content (*F*_(2, 492)_ = 0.019, *p* = 0.981) were not significant, indicating that species-specific differences in survival changes over time were more strongly associated with moisture conditions than with temperature effects alone.

The four-way interaction of species × temperature × moisture content × storage duration was highly significant (*F*_(227, 456)_ = 17.923, *p* < 0.001). This result indicates that the temporal patterns of nematode survival depended on the combined effects of species identity, temperature, and moisture conditions, with clear species-specific differences in survival responses under different storage regimes.

Collectively, these results demonstrate that nematode storage survival is shaped by multiple interacting factors, with storage duration, moisture content, temperature, and species identity jointly influencing both the overall level of survival and its temporal trajectory. The specific manifestations of these interactions in the survival dynamics of the two nematode species are elaborated in detail in the following sections.

### 3.2. Dynamic Changes in Survival Under the Interacting Effects of Temperature, Moisture Content, and Species

Given that storage duration significantly affected survival (Section 3.1), the following analyses examine how temperature, moisture content, and nematode species influenced survival patterns over time.

#### 3.2.1. Temperature Strongly Influences Long-Term Survival

**Figure 2:**
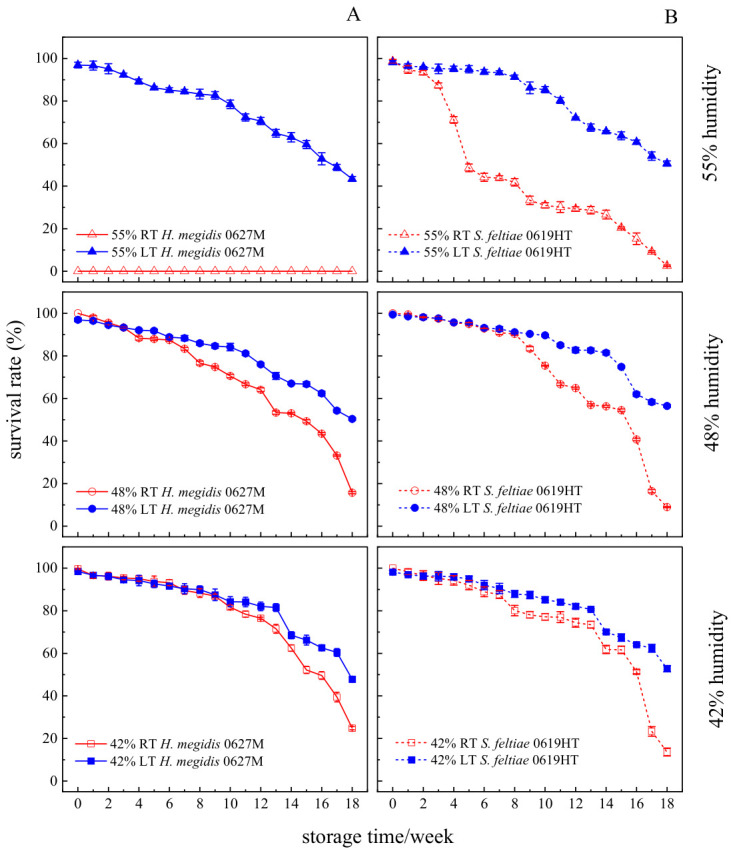
Temporal dynamics of survival rates during 18-week storage for *H. megidis* 0627M (panel (**A**)) and *S. feltiae* 0619HT (panel (**B**)) under different combinations of temperature and moisture content. Data points represent mean survival rates. Different marker shapes and colors denote the temperature treatments. RT: room temperature (25 °C); LT: low temperature (6 °C).

Among the environmental factors tested, temperature strongly influenced the temporal trajectory of survival decline. Across the 18-week storage period, low-temperature storage (6 °C) consistently delayed the decline in survival for both entomopathogenic nematode species across all moisture content levels (Figure 2). This temperature effect became particularly evident after approximately four weeks of storage and persisted until the end of the experiment. In contrast, survival under room temperature (25 °C) declined more rapidly, indicating that temperature conditions significantly affected the temporal dynamics of nematode viability during storage.

#### 3.2.2. Species-Specific Responses Under Low-Temperature Storage

*Steinernema feltiae* 0619HT exhibited higher tolerance to low-temperature storage. Under low-temperature conditions, its survival rate was consistently higher than that of *H. megidis* 0627M across most moisture content treatments (Figure 2B). Notably, at 48% moisture content—the moisture level associated with the highest survival within the tested range—its survival rate remained above 50% after 18 weeks of storage, showing significantly higher survival than the other tested conditions (Figure 2). In contrast, under room-temperature storage at 25 °C, the survival rate of *S. feltiae* 0619HT declined rapidly across all moisture content levels, indicating poor survival performance under prolonged room-temperature storage conditions.

#### 3.2.3. Delayed Survival Advantage of *H. megidis* 0627M Under Room-Temperature Storage

In contrast to the *S. feltiae* 0619HT, *H. megidis* 0627M exhibited a distinct temporal response pattern under room-temperature storage. At 25 °C, it showed improved survival performance under lower moisture conditions over prolonged storage. At 42% moisture content, its survival rate significantly surpassed that of *S. feltiae* 0619HT starting from week 8 (week 8: *H. megidis* 0627M 88.32% vs. S. *feltiae* 0619HT 80.11%; *F*_(37, 76)_ = 5.07, *p* < 0.01). At 48% moisture content, despite a lower initial survival rate, its survival rate reached 43.16% at approximately week 16, significantly exceeding that of *S. feltiae* (40.74%) and maintaining this advantage until the end of the experiment (*F*_(37, 76)_ = 54.28, *p* < 0.05) (Figure 2A). This time-dependent shift in survival performance highlights interspecific differences in tolerance to prolonged combined thermal and moisture stress between the two nematode species.

#### 3.2.4. Temperature-Dependent Effects of Moisture Content on Survival

Over the 18-week storage period, survival patterns differed significantly among temperature–moisture combinations. Low-temperature storage resulted in higher survival under the tested moisture conditions. *Steinernema feltiae* 0619HT achieved the highest survival at the end of the storage period under 6 °C and 48% moisture content, with 56.48% survival at week 18. The next best-performing treatments were *S. feltiae* 0619HT (52.79% survival) and *H. megidis* 0627M (47.85% survival), both under 6 °C and 42% moisture content, indicating that the combination of low temperature and moderate-to-low moisture content was associated with improved long-term survival of EPNs.

Under room-temperature conditions (25 °C), survival rates of both species declined progressively with increasing moisture content. The lowest moisture level (42%) resulted in the highest survival rates, whereas the highest mortality was recorded at 55% moisture content. Notably, *S. feltiae* 0619HT at 25 °C and 55% moisture content exhibited near-complete mortality by week 18 (survival rate < 3%), while *H. megidis* 0627M showed complete mortality from the beginning of storage. These results indicate that high moisture conditions at elevated temperature were associated with substantially reduced long-term survival of both nematode species.

Importantly, all low-temperature treatment groups exhibited consistently higher survival rates than their corresponding room-temperature counterparts at equivalent moisture content levels. These results indicated that temperature was the strongest influencing factor affecting long-term nematode survival, while moisture content represented an additional important factor influencing storage performance (Figure 2).

#### 3.2.5. Integrated Assessment of Storage Performance

**Figure 3:**
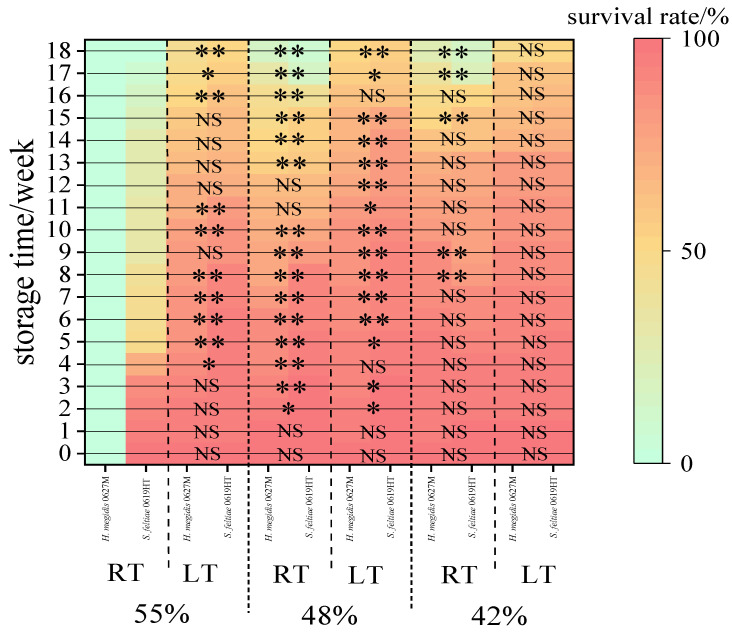
Survival Rates of Different Nematode Species under Various Storage Conditions over Time. Note: 55%, 48%, and 42% represent moisture content levels; RT denotes room temperature, LT denotes low temperature. The color gradient from red to green indicates nematode survival rates from high to low. Symbols denote differences between temperatures at the same time point: *: Significant difference (*p* < 0.05); **: Highly significant difference (*p* < 0.01); NS: No significant difference (*p* > 0.05).

To visualize how different storage conditions affected long-term storage performance, a heatmap analysis was employed to display the species-specific temporal dynamics under various temperature and moisture content combinations (Figure 3).

Under low-temperature storage (6 °C) at 55% moisture content, *S. feltiae* 0619HT consistently maintained higher survival rates than *H. megidis* 0627M throughout most of the storage period. With the exception of weeks 0–3, week 9, and weeks 12–15, where no significant differences were detected (*p* > 0.05), and weeks 4 and 17, where moderately significant differences were observed (*p* < 0.05), interspecific differences were highly significant at the majority of time points (*p* < 0.01, *F*_(37, 76)_ = 1.79). At 48% moisture content, *S. feltiae* 0619HT exhibited higher survival than *H. megidis* 0627M at most time points. Interspecific differences were non-significant at weeks 0–1, 4, and 16 (*p* > 0.05), significant at weeks 2, 3, 5, 11, and 17 (*p* < 0.05), and highly significant at all remaining time points (*p* < 0.01, *F*_(37, 76)_ = 7.12). At 42% moisture content, no significant interspecific differences were detected at any time point (*p* > 0.05), indicating similar survival performance between the two species under low-moisture, low-temperature storage conditions.

Under room-temperature storage (25 °C) at 48% moisture content, a time-dependent shift in survival performance was observed between the two species. *Steinernema feltiae* 0619HT exhibited higher survival during the early storage period (weeks 0–15), after which *H. megidis* 0627M gradually gained the advantage. By week 16, the survival rate of *H. megidis* 0627M significantly exceeded that of *S. feltiae* 0619HT (43.16% vs. 40.74%; *p* < 0.01). Apart from weeks 0, 1, 11, and 12, where differences were non-significant (*p* > 0.05), and week 2, where differences were significant (*p* < 0.05), interspecific differences were highly significant at the majority of time points (*p* < 0.01, *F*_(37, 76)_ = 54.28). At 42% moisture content, *H. megidis* 0627M showed higher survival than *S. feltiae* 0619HT with increasing storage duration. Except for weeks 0–7, 10–14, and 16, where survival rates were comparable (*p* > 0.05), differences were highly significant at most time points (*p* < 0.01, *F*_(37, 76)_ = 5.07). At 55% moisture content, both species exhibited rapid mortality, with *H. megidis* 0627M showing complete mortality from the beginning of storage, whereas the survival rate of *S. feltiae* 0619HT declined to 2.58% by week 18.

After 18 weeks of storage, the most favorable conditions within the tested range differed markedly between the two temperature regimes (Figure 3). Under room temperature (25 °C), survival rates of both species declined with increasing moisture content, with 42% moisture content being associated with the highest survival rates and highly significant interspecific differences (*S. feltiae* 0619HT: 13.62%; *H. megidis* 0627M: 24.84%; *p* < 0.01). Both species showed severe survival decline under 55% moisture content at room temperature. Under low temperature (6 °C), survival reached the highest level within the tested moisture range at 48% moisture content, with highly significant interspecific differences (*S. feltiae* 0619HT: 56.48%; *H. megidis* 0627M: 50.37%; *p* < 0.01), while the lowest survival was recorded at 55% moisture content, also with highly significant differences (*S. feltiae* 0619HT: 50.61%; *H. megidis* 0627M: 43.39%; *p* < 0.01).

Collectively, these results indicate that storage conditions associated with higher survival differed between species and depended on the interactions among temperature, moisture content, and storage duration. *Steinernema feltiae* 0619HT showed the highest survival under low-temperature and moderate-moisture conditions, whereas *H. megidis* 0627M maintained higher survival under prolonged room-temperature storage with low moisture content.

### 3.3. Quantitative Assessment of Mortality Risk Using Generalized Linear Mixed Model (GLMM)

To quantify the relative contribution of storage parameters to nematode mortality risk, a GLMM was constructed using species, temperature, and moisture content as explanatory variables (Table 2). Temperature was identified as the strongest influencing factor affecting mortality risk. Storage at 6 °C significantly reduced the odds of death by 82.4% compared with room-temperature storage (25 °C; odds ratio [OR] = 0.176, 95% CI: 0.117–0.263, *p* < 0.001), highlighting the critical role of low temperature in maintaining nematode survival during storage. Moisture content also exerted a significant influence on survival risk. Using 48% moisture content as the reference level, high moisture content (55%) increased the odds of mortality by more than 9-fold (OR = 10.114, 95% CI: 6.155–16.618, *p* < 0.001), whereas no significant difference in hazard was detected between 42% and 48% moisture content (*p* = 0.810). These results indicate that excessive moisture was associated with increased mortality risk during storage, whereas moderate reductions in moisture content did not substantially affect survival. Species identity had a significant but comparatively smaller effect on mortality risk. Across all storage conditions, *S. feltiae* 0619HT exhibited an approximately 67.2% lower overall odds of death than *H. megidis* 0627M (OR = 0.328, 95% CI: 0.219–0.490, *p* < 0.001), indicating a higher survival probability of this species under the tested storage conditions. Collectively, the GLMM revealed a relative importance pattern among storage-related factors, with low temperature (6 °C) providing the strongest protective effect, followed by the intrinsic tolerance of the *S. feltiae* strain, while excessive moisture (55%) acts as the most critical risk factor.

### 3.4. Effects of Storage Conditions on Nematode Virulence After Long-Term Storage

**Figure 4:**
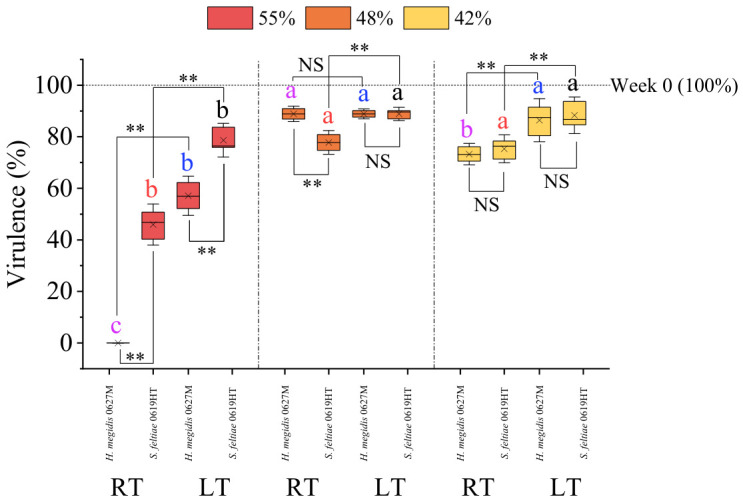
Virulence of Different Nematode Species under Different Storage Conditions at 18 Weeks. Asterisks (**) and NS indicate significant differences in virulence between different species at the same temperature and the same species at different temperatures: ** for highly significant (*p* < 0.01), and NS for not significant (*p* > 0.05). Different lowercase letters in different colors indicate significant differences between different moisture content levels for the same nematode species at the same temperature (one-way ANOVA, *p* < 0.05). The horizontal dashed line at 100% represents the week 0 virulence of freshly harvested IJs (≤5 days post-emergence).

After 18 weeks of storage, virulence assays showed that storage conditions affected not only nematode survival rates but also the infective capacity of surviving IJs (Figure 4; Table 3).

**Interspecific differences in virulence across moisture levels**

At 55% moisture content, *S. feltiae* 0619HT exhibited significantly higher virulence than *H. megidis* 0627M under both room-temperature (*F*_(5, 12)_ = 128.53, *p* < 0.01) and low-temperature (*F*_(5, 12)_ = 12.52, *p* < 0.01) storage conditions. Furthermore, both species showed significantly higher virulence after low-temperature storage than after room-temperature storage (*H. megidis* 0627M: *F*_(5, 12)_ = 114.73, *p* < 0.01; *S. feltiae* 0619HT: *F*_(5, 12)_ = 13.62, *p* < 0.01).

At 48% moisture content, interspecific differences in virulence varied between temperature regimes. Under room-temperature storage, the virulence of *H. megidis* 0627M was significantly higher than that of *S. feltiae* 0619HT (*F*_(5, 12)_ = 128.53, *p* < 0.01), whereas no significant interspecific difference was detected after low-temperature storage (*p* > 0.05). Species-specific responses to storage temperature were observed in the intraspecific comparisons: the virulence of *H. megidis* 0627M was not significantly affected by storage temperature (*p* > 0.05), whereas that of *S. feltiae* 0619HT was significantly higher after low-temperature storage than after room-temperature storage (*F*_(5, 12)_ = 13.62, *p* < 0.01).

At 42% moisture content, no significant interspecific differences in virulence were detected under either temperature regime (*p* > 0.05). Nevertheless, low-temperature storage resulted in significantly higher retained virulence in both species compared with room-temperature storage (*H. megidis* 0627M: *F*_(5, 12)_ = 114.73, *p* < 0.01; *S. feltiae* 0619HT: *F*_(5, 12)_ = 13.62, *p* < 0.01).

**Intraspecific effects of moisture content on virulence**

For *H. megidis* 0627M under room-temperature storage, virulence varied significantly among moisture levels (*F*_(2, 6)_ = 1720.06, *p* < 0.01), with the highest virulence recorded at 48% moisture content and the lowest at 55%. Under low-temperature storage, no significant difference in virulence was detected between 42% and 48% moisture content (*p* > 0.05); however, both levels resulted in significantly higher virulence than 55% moisture content. For *S. feltiae* 0619HT, virulence at 42% and 48% moisture content did not differ significantly under either room-temperature or low-temperature storage (*p* > 0.05), whereas both moisture levels maintained significantly higher virulence than at 55% moisture content.

Collectively, with the exception of room-temperature storage at 48% moisture content—where *H. megidis* 0627M exhibited significantly higher virulence than *S. feltiae* 0619HT and was unaffected by storage temperature—*S. feltiae* 0619HT showed higher virulence in most other treatment combinations. Notably, low-temperature storage resulted in better retention of virulence in both species under most moisture content conditions, indicating that cold storage not only improves survival retention but also more effectively preserves nematode infective after long-term storage.

**Relationship between endpoint survival rate and virulence**

The analysis indicated that at the storage endpoint (week 18), survival rate and virulence were not consistently concordant across treatment combinations, indicating a descriptive relationship rather than a statistically validated correlation. For example, under low-temperature storage at 48% moisture content and room-temperature storage at 42% moisture content, no significant interspecific differences in virulence were detected despite highly significant differences in survival rate at week 18 (*p* < 0.01) (Figure 3). At 42% moisture content under low temperature, both survival rates (52.79% vs. 47.85%) and virulence were comparable between the two species (Figure 3). Conversely, at 55% moisture content under low temperature, the significantly higher survival rate of *S. feltiae* 0619HT (50.61%) relative to *H. megidis* 0627M (43.39%) was accompanied by significantly greater virulence. Similarly, under room-temperature storage at 48% moisture content, the higher survival rate of *H. megidis* 0627M (15.74%) compared with *S. feltiae* 0619HT (8.97%) was associated with significantly higher virulence. These results suggest that survival and virulence are distinct and complementary metrics, and both should be evaluated when optimizing long-term storage conditions to ensure not only nematode viability but also functional infectivity.

## 4. Discussion

This study systematically evaluated the long-term storage potential of two indigenous EPNs from Gansu Province, China—*H. megidis* 0627M and *S. feltiae* 0619HT—under different storage temperatures and substrate moisture conditions. The results demonstrated that in sponge carriers, both survival rate and post-storage virulence were strongly shaped by the interaction between temperature and moisture content, with different species exhibiting distinct storage response patterns. Low-temperature storage markedly reduced mortality risk and more effectively preserved post-storage infectivity across most conditions, whereas the most favorable moisture levels within the tested range varied depending on both temperature and nematode species. Survival rate and virulence did not consistently co-vary across storage conditions.

Our findings demonstrate that low temperature (6 °C) significantly prolonged the survival of both nematode species, consistent with the well-established principle that low temperatures can reduce metabolic activity and help conserve energy reserves in EPNs [49,57,58,59]. *Steinernema feltiae* 0619HT maintained a survival rate of 56.48% after 18 weeks of storage at 6 °C and 48% moisture content in polyurethane sponge—a result broadly comparable to the approximately 55% survival reported by Touray et al. [49] for an *S. feltiae* strain after 8 months at 10 °C in Scotchbrite sponge. Under low-temperature conditions, *S. feltiae* 0619HT consistently maintained higher survival rates than *H. megidis* 0627M, indicating greater tolerance of this species to cold storage conditions. This disparity may be attributable to interspecific differences in energy reserve composition and physiological traits associated with cold tolerance. Previous studies have reported that *Steinernema* possesses relatively high lipid reserves; compounds such as glycerol may serve not only as energy sources during prolonged quiescence but also as membrane stabilizers and cryoprotectants under low-temperature conditions [60]. Furthermore, cold exposure has been shown to rapidly induce the accumulation of protective carbohydrates such as trehalose in this species [61], as well as upregulate the expression of stress-resistance proteins, including heat shock proteins (HSPs) and late embryogenesis abundant (LEA) proteins [62]. These coordinated molecular responses may contribute to the maintenance of cellular structural integrity and improved survival under refrigerated storage conditions.

In contrast, *H. megidis* 0627M showed a delayed storage advantage under room-temperature storage with low moisture content. Specifically, at 25 °C, this species outperformed *S. feltiae* 0619HT from week 8 onward at 42% moisture content, and from week 16 onward at 48% moisture content. This finding differs from the general pattern that *Heterorhabditis* spp. are less tolerant than *Steinernema* spp. under ambient-temperature storage conditions [21]. We propose that this strain-specific response may be associated with local environmental adaptation and differential stress tolerance traits; under prolonged moisture stress at room temperature, *H. megidis* 0627M may gradually activate protective responses, including the accumulation of late embryogenesis abundant (LEA) proteins, trehalose, and glycerol. It has been reported that these protective compounds can be induced in response to chronic rather than acute desiccation stress [63,64]. The time-dependent nature of this competitive reversal is consistent with this hypothesis: a stress-response process requiring progressive induction would be expected to confer advantage only after sufficient storage duration, rather than from the outset. However, this mechanistic explanation remains speculative. Future studies should quantify trehalose accumulation kinetics and LEA protein expression levels across storage time points to directly test this hypothesis.

Significant interspecific differences in stress tolerance exist among EPNs [38], and temperature and moisture content are known to exert closely coupled interactive effects on nematode survival [51]. Our study revealed that although survival rates of both EPN species increased with decreasing moisture content, the most favorable moisture level within the tested range exhibited a marked temperature dependence. After 18 weeks of storage, 48% moisture content provided the most favorable survival conditions for both species under low-temperature storage. This specific combination may have balanced water availability and desiccation stress, thereby helping maintain energy reserves in both species—particularly *S. feltiae* 0619HT—under a low-metabolic state. Conversely, under room-temperature conditions, 42% moisture content proved most favorable within the tested range. The reduced moisture level imposed greater desiccation pressure, forcing nematodes into a deeper state of quiescence and substantially decelerating the rate of energy depletion [50], which conferred a pronounced survival advantage to the drought-adapted strain *H. megidis* 0627M.

Notably, *H. megidis* 0627M underwent complete mortality within 24 h under room-temperature storage with 55% moisture content, a result that was consistent across all three biological replicate tubes (n = 3), accompanied by a distinctive odor upon tube opening. The mechanism underlying such rapid and complete mortality under warm, high-humidity conditions remains to be elucidated. A plausible explanation is rapid microbial proliferation within the sponge matrix: at 25 °C and 55% moisture, the environment simultaneously favors microbial growth and increases the metabolic demands of nematodes. Under these conditions, the balance between IJs’ energy reserves and microbial stress may rapidly shift toward mortality, particularly in *Heterorhabditis* spp., which possess a relatively narrow thermal niche and are better adapted to cooler environments [64]. Upon opening these storage tubes, we noticed a distinctive odor consistent with microbial decomposition of nematode biomass. Although this observation has not been experimentally confirmed, the odor may involve volatile amine compounds such as cadaverine and putrescine, which are known to function as necromone signals in some nematode systems [65,66]. Direct future investigations—including microbiome profiling and quantitative analysis of volatile organic compounds—are required to determine the specific mechanisms.

Survival rate and virulence are not equivalent parameters; storage conditions affect not only the quantity of viable IJs but also their functional quality [49,67]. Following 18 weeks of room-temperature storage, even surviving IJs exhibited diminished host-seeking and infectivity capabilities, a consequence of nematode aging during prolonged storage [68]. Our results demonstrate that low temperature effectively maintained virulence under the majority of conditions tested, consistent with previous findings [57,59]. The underlying protective mechanism may involve reduced oxidative metabolism and decreased reactive oxygen species (ROS) accumulation under low-temperature conditions, thereby mitigating degenerative damage to virulence-associated molecular systems. Under most low-temperature conditions, *S. feltiae* 0619HT exhibited significantly higher virulence than *H. megidis* 0627M, which may reflect species-specific cold-tolerance traits associated with *Steinernema,* including its interactions with symbiotic bacteria (*Xenorhabdus* spp.) and chemotaxis mechanisms, consistent with previously documented cold-tolerance traits of this genus [60,61,62,69]. In contrast, storage temperature had no significant effect on the virulence of *H. megidis* 0627M at room temperature and 48% moisture content; moreover, under these specific conditions, its virulence was significantly higher than that of *S. feltiae* 0619HT. This finding suggests that prolonged storage does not inevitably compromise nematode virulence when conditions are appropriately matched to species physiology [46], and that different species may possess species-specific optima for virulence retention [70,71]. For *H. megidis* 0627M, these conditions may more closely resemble its native environmental conditions, enabling a favorable balance between energy expenditure and the maintenance of infective capacity. The VRI summarized in Table 3 further quantitatively supports this conclusion, demonstrating a consistent pattern across the tested storage regimes. Collectively, these observations suggest that survival rate alone may not be a sufficient indicator of storage quality under all conditions, and that virulence should be evaluated as a complementary metric alongside survival—particularly in treatment combinations where IJs remain viable but may experience functional deterioration.

In long-term storage systems for EPNs, the selection of storage substrate is a critical factor because the carrier directly influences EPN survival and biological activity [63,72]. In the present study, sponge was employed as a storage carrier and demonstrated practical advantages in terms of handling convenience, availability, and the maintenance of nematode viability under appropriate moisture and temperature conditions, consistent with findings reported by Touray et al. [49] using comparable absorbent substrates. However, nematode retention within the sponge matrix may result in reduced recovery efficiency during field application [73]; future formulation improvements could incorporate desiccation protectants or composite moisture-retaining materials to enhance storage efficiency [74,75]. At the species selection level, although abiotic factors undoubtedly shape EPN storage performance and field efficacy [20], the selection of indigenous species with strong tolerance to local climatic conditions remains a fundamental prerequisite for improving formulation stability and biocontrol effectiveness. The efficacy of indigenous EPNs generally exhibits region-specific variation [21], a pattern consistently validated across multiple countries and regions [8,35,52,76]. The *H. megidis* 0627M and *S. feltiae* 0619HT used in this study were isolated from Tianshui City and Baiyin City in Gansu Province, respectively, and have been demonstrated to possess strong adaptability to their local environments [8], providing a valuable resource basis for their further development as regional biological control products.

While this study systematically evaluated the effects of temperature and moisture content on the storage performance of two indigenous EPN species, several limitations warrant acknowledgment. First, adequate ventilation is a critical factor influencing the long-term survival of EPNs under room-temperature storage [77]. As containers were necessarily opened weekly for sampling, the experimental conditions did not fully represent a commercially sealed packaging environment; whether EPNs can maintain viability for 18 weeks or longer under completely sealed conditions, therefore, remains to be verified. Relatedly, repeated opening of the same tube throughout the storage period may have introduced minor cumulative perturbations to the tube microenvironment, including potential changes in oxygen levels, moisture distribution, and microbial dynamics. Although within-tube correlation was statistically accounted for by including Subject ID as a random intercept in the GLMM, the potential effects of repeated tube opening on nematode survival cannot be completely excluded. It should also be noted that the moisture-related conclusions of this study are bounded by the specific range tested (42–55% VMC) and should not be interpreted as representing a global optimum across the full theoretical range of substrate moisture content; further investigation across a broader moisture gradient would be required to determine whether more favorable conditions exist outside this range. Similarly, no non-sponge control (e.g., aqueous suspension storage) was included in the present design; the findings should therefore be interpreted as characterizing the optimization of storage conditions within the sponge-based system itself, consistent with the cost-effectiveness and operational simplicity that motivated its selection as a carrier material in this study [49]. Direct comparison with alternative carrier materials, while beyond the scope of the present study, represents a valuable direction for future research. Second, key stress-resistance physiological indicators—such as trehalose accumulation and heat shock protein expression—were not dynamically monitored throughout the storage period, precluding mechanistic elucidation of the observed survival differences at the molecular level. Third, virulence was assessed exclusively at week 18; therefore, the temporal dynamics of virulence changes throughout the storage period remain uncharacterized. Furthermore, the present study examined only two species and three moisture content levels, and the generalizability of these conclusions requires validation through broader multi-species comparisons and more refined parameter optimization. Future research should integrate physiological, biochemical, and omics-based approaches to further elucidate the energy metabolism and stress response mechanisms of EPNs during prolonged storage, and to explore optimized storage strategies under fully sealed conditions or multi-factor synergistic regimes, such as oxygen regulation and composite moisture-retention systems.

In summary, this study demonstrated the advantage of low-temperature storage for *S. feltiae* 0619HT and characterized the delayed storage superiority of *H. megidis* 0627M under room-temperature and low-moisture conditions. Through moisture content regulation in sponge carriers, species-specific storage strategies were developed for both indigenous EPN species, providing quantifiable laboratory parameters to support the future optimization and transportation of commercial EPN formulations. Notably, *S. feltiae* 0619HT achieved 56.48% survival after 18 weeks at 6 °C, broadly consistent with survival rates reported for *S. feltiae* stored in sponge substrates under comparable refrigerated conditions [49], while *H. megidis* 0627M demonstrated a distinct virulence advantage under room-temperature storage at 48% moisture content—conditions that may better reflect the environmental characteristics of arid northwestern China. These results suggest that locally adapted indigenous strains can achieve comparable performance to reference strains under cold-chain conditions while providing complementary advantages under ambient low-moisture storage, highlighting their potential for the development of regionally targeted and cost-effective biocontrol formulations. These findings indicate that the implementation of differentiated, species-specific storage strategies represents an important approach for maintaining both nematode viability and virulence, thereby improving the storage stability of EPN-based biocontrol products. Looking ahead, further enhancement of EPN tolerance to complex environments—such as arid and semi-arid regions—should be pursued through the integration of optimized carrier formulations, species genetic improvement, and innovations in formulation technology, collectively advancing the large-scale application of EPNs in regionally sustainable green agriculture.

## Figures and Tables

**Table 1:** Tests of fixed effects on nematode survival rate from the generalized linear mixed model.

Source	Num df	Den df	*F*	*p*
Storage Time	18	661	8.683	<0.001
Moisture	2	661	56.624	<0.001
Species	1	661	29.36	<0.001
Temperature	1	661	71.139	<0.001
Temperature × Moisture	2	584	100.883	<0.001
Species × Temperature	1	584	38.005	<0.001
Species × Moisture	2	584	44.733	<0.001
Temperature × Storage Time	18	584	0.833	0.662
Species × Storage Time	18	584	0.54	0.939
Moisture × Storage Time	36	584	0.204	1.000
Species × Temperature × Moisture	2	492	0.019	0.981
Species × Temperature × Storage Time	18	492	1.534	0.074
Species × Moisture × Storage Time	36	492	1.538	0.026
Temperature × Moisture × Storage Time	36	492	2.143	<0.001
Species × Temperature × Moisture × Storage Time	227	456	17.923	<0.001

Note. Results are based on Type III F-tests for fixed effects from a generalized linear mixed-effects model with a binomial distribution and logit link function. Subject ID was included as a random intercept to account for repeated measurements. Degrees of freedom were estimated using the Satterthwaite approximation.

**Table 2:** Parameter estimates and odds ratios from the generalized linear mixed model for the survival of two EPN species.

Variable	Comparison	B	SE	Wald	*p*-Value	Exp(B) (OR)	95% CI for Exp(B)	
							LL	UL
Species	*S. feltiae* 0619HT vs. *H. megidis* 0627M (Ref)	−1.116	0.206	−5.432	<0.001	0.328	0.219	0.49
Temperature	6 °C vs. 25 °C (Ref)	−1.738	0.206	−8.459	<0.001	0.176	0.117	0.263
Moisture content	55% vs. 48% (Ref)	2.314	0.253	9.15	<0.001	10.114	6.155	16.618
	42% vs. 48% (Ref)	−0.06	0.25	−0.241	0.810	0.942	0.577	1.537

**Note:** The model used a binomial distribution with a logit link function, with DeadCount/TotalCount as the target variable. (Ref.) indicates the reference category for each variable. Exp(B) represents the odds ratio (OR). 95% CI represents the 95% confidence interval for the odds ratio. *p*-values < 0.05 were considered statistically significant.

**Table 3:** Virulence and VRI of *H. megidis* 0627M and *S. feltiae* 0619HT after 18 weeks of storage under different conditions.

Moisture	Temperature	Species	*n*	Mean Mortality (%) ± SD
55%	Room Temperature	*H. megidis* 0627M	3	0.00 ± 0.00
55%	Room Temperature	*S. feltiae* 0619HT	3	45.94 ± 5.32
55%	Low Temperature	*H. megidis* 0627M	3	57.10 ± 5.07
55%	Low Temperature	*S. feltiae* 0619HT	3	78.66 ± 4.35
48%	Room Temperature	*H. megidis* 0627M	3	88.89 ± 2.01
48%	Room Temperature	*S. feltiae* 0619HT	3	77.80 ± 3.07
48%	Low Temperature	*H. megidis* 0627M	3	88.89 ± 1.29
48%	Low Temperature	*S. feltiae* 0619HT	3	88.89 ± 1.70
42%	Room Temperature	*H. megidis* 0627M	3	73.26 ± 2.78
42%	Room Temperature	*S. feltiae* 0619HT	3	75.33 ± 3.61
42%	Low Temperature	*H. megidis* 0627M	3	86.44 ± 5.57
42%	Low Temperature	*S. feltiae* 0619HT	3	88.34 ± 4.73
Control	—	Fresh Ijs	3	100.00 ± 0.00
No-nematode control	—	—	3	0.00 ± 0.00

**Note:** Values represent the mean mortality ± standard deviation (SD) from three biological replicates per treatment. Each replicate contained 6 larvae, with a total of 18 larvae tested per treatment group. The Week 0 Control (freshly emerged IJs) exhibited 100.00 ± 0.00% mortality, and its VRI was set to 100. The no-nematode control group consistently showed 0.00% mortality across all experiments. Because week-0 virulence of freshly emerged IJs was 100%, VRI values are numerically identical to week-18 mean mortality values and are therefore omitted as a separate column. “—” indicates not applicable.

## Data Availability

The datasets in this study are available from the corresponding author on reasonable request.

## Supplementary Materials

The following supporting information can be downloaded at: https://www.mdpi.com/article/10.3390/insects17070723/s1, Table S1: Summary of the full experimental design.
