# Supplementary material for: Effects of Temperature-Moisture Interactions on Storage Survival and Virulence in Two Entomopathogenic Nematode Species
Source: Insects. 2026 Jul 13;17(7):723. doi: 10.3390/insects17070723 (PMC13409990; doi:10.3390/insects17070723)
Supplement: Supplementary file 1 [file insects-17-00723-s001.zip › insects-4343863-supplementary.pdf]

Table S1. Summary of the full experimental design

Factor	Levels	Details
Species	2	*Heterorhabditis megidis* 0627M; *Steinernema feltiae* 0619HT
Temperature	2	6°C (LT); 25°C (RT)
Moisture content	3	42%; 48%; 55% VMC
Treatment combinations	12	2 × 2 × 3
Tubes per treatment combination	3	Independent biological replicates
Total tubes	36	12 combinations × 3 tubes
Sampling time points	19	Week 0 to Week 18 (weekly intervals)
Sampling method	Repeated measures	One sponge block (~0.5 cm^3) removed per tube per time point; block discarded after counting
Sponge blocks per time point	36	12 combinations × 3 tubes
Total sponge blocks sampled	684	36 blocks × 19 time points
IJs counted per sample	≥100	All available IJs counted when recovery < 100
Statistical unit	Individual tube	Included as random intercept (Subject ID) in GLMM
